# Supplementary material for: Resolving the full spectrum of human genome variation using Linked-Reads
Source: Genome Res. 2019 Apr;29(4):635–45. doi: 10.1101/gr.234443.118 (PMC6442396; doi:10.1101/gr.234443.118)
Supplement: Supplemental Material [file supp_gr.234443.118_Supplemental_longranger-2.2.zip › longranger-2.2.tar.bz2!/lr2.2-public/lib/stan/cmdstan/stan/doc/api/html/functions_2promote__scalar_8hpp_source.html]

Stan: src/stan/math/functions/promote\_scalar.hpp Source File


|  |  |
| --- | --- |
|  | Stan  2.5.0  probability, sampling & optimization |


- Main Page
- Related Pages
- Namespaces
- Classes
- Files

- File List
- File Members

All Classes Namespaces Files Functions Variables Typedefs Enumerations Enumerator Friends Macros Pages

- src
- stan
- math
- functions

promote\_scalar.hpp

Go to the documentation of this file.

1 #ifndef STAN\_\_MATH\_\_FUNCTIONS\_\_PROMOTE\_SCALAR\_HPP

2 #define STAN\_\_MATH\_\_FUNCTIONS\_\_PROMOTE\_SCALAR\_HPP

3

4 #include <vector>

5 #include <stan/math/functions/promote\_scalar\_type.hpp>

6 #include <stan/math/meta/index\_type.hpp>

7

8 namespace stan {

9

10  namespace math {

11

20  template <typename T, typename S>

21  struct promote\_scalar\_struct {

22

34  static T apply(S x) {

35  return T(x);

36  }

37  };

38

46  template <typename T>

47  struct promote\_scalar\_struct<T,T> {

48

55  static T apply(const T& x) {

56  return x;

57  }

58  };

59

69  template <typename T, typename S>

70  struct promote\_scalar\_struct<T, std::vector<S> > {

71

80  static std::vector<typename promote\_scalar\_type<T,S>::type>

81  apply(const std::vector<S>& x) {

82  typedef std::vector<typename promote\_scalar\_type<T,S>::type> return\_t;

83  typedef typename index\_type<return\_t>::type idx\_t;

84  return\_t y(x.size());

85  for (idx\_t i = 0; i < x.size(); ++i)

86  y[i] = promote\_scalar\_struct<T,S>::apply(x[i]);

87  return y;

88  }

89

90  };

91

101  template <typename T, typename S>

102  typename promote\_scalar\_type<T,S>::type

103  promote\_scalar(const S& x) {

104  return promote\_scalar\_struct<T,S>::apply(x);

105  }

106

107

108  }

109 }

110

111 #endif

stan::math::promote\_scalar\_struct< T, std::vector< S > >::apply

static std::vector< typename promote\_scalar\_type< T, S >::type > apply(const std::vector< S > &x)

Return the standard vector consisting of the recursive promotion of the elements of the input standar...

**Definition:** promote\_scalar.hpp:81

stan::math::promote\_scalar\_struct< T, T >::apply

static T apply(const T &x)

Return the unmodified input.

**Definition:** promote\_scalar.hpp:55

promote\_scalar\_type.hpp

stan::math::index\_type

Primary template class for the metaprogram to compute the index type of a container.

**Definition:** index\_type.hpp:21

stan::math::promote\_scalar\_struct

General struct to hold static function for promoting underlying scalar types.

**Definition:** promote\_scalar.hpp:21

stan::math::promote\_scalar\_struct::apply

static T apply(S x)

Return the value of the input argument promoted to the type specified by the template parameter...

**Definition:** promote\_scalar.hpp:34

stan::math::promote\_scalar

promote\_scalar\_type< T, S >::type promote\_scalar(const S &x)

This is the top-level function to call to promote the scalar types of an input of type S to type T...

**Definition:** promote\_scalar.hpp:103

index\_type.hpp


---

[ Stan Home Page ]

*© 2011–2014,
Stan Development Team.*
